# Amino acid‐induced impairment of insulin sensitivity in healthy and obese rats is reversible

**DOI:** 10.14814/phy2.12067

**Published:** 2014-07-04

**Authors:** Senthure Jeganathan, Abdikarim Abdullahi, Sana Zargar, Naomi Maeda, Michael C. Riddell, Olasunkanmi A. J. Adegoke

**Affiliations:** 1 School of Kinesiology and Health Science and Muscle Health Research Centre, York University, Toronto, Ontario, Canada

**Keywords:** Glucose metabolism, insulin resistance, leucine, skeletal muscle

## Abstract

High‐protein diets (HPDs) promote weight loss but other studies implicate these diets and their constituent amino acids (AAs) in insulin resistance. We hypothesized that AA‐induced insulin resistance is a temporal and reversible metabolic event. L6 myotubes were serum deprived for 4 h and then incubated in AA and/or insulin (100 nmol/L). Another group of cells was incubated overnight in AA + insulin, starved again, and then reincubated with AA and insulin. Mammalian (mechanistic) target of rapamycin complex 1 (mTORC1) signaling and glucose uptake were then measured. Healthy or insulin‐resistant rats were gavaged with leucine (0.48 g/kg) and insulin sensitivity was examined. In myotubes, incubation with AA and insulin significantly (*P *<**0.05) increased the phosphorylation of the mTORC1 substrate ribosomal protein S6 kinase 1 (S6K1, T389) and of insulin receptor substrate 1 (IRS‐1, serine residues), but suppressed insulin‐stimulated glucose uptake by 40% (*P *<**0.01). These modifications were mTORC1‐dependent and were reversible. In vivo, leucine gavage reversibly increased S6K1 phosphorylation and IRS‐1 serine phosphorylation 5‐ to 12‐fold in skeletal muscle and impaired insulin tolerance of glucose (*P *<**0.05) in lean rats. In insulin‐resistant rats, the impairment of whole blood glucose and AA metabolism induced by leucine gavage (0.001 < *P *<**0.05) was more severe than that observed in lean rats; however, the impairment was reversible within 24 h of treatment. If these data are confirmed in long‐term studies, it would imply that the use of leucine/HPD in treating metabolic diseases is unlikely to have lasting negative effects on insulin sensitivity.

## Abstract (precis)

Using in vitro and in vivo systems, we show that amino acid‐induced modulation of mTORC1/S6K1 signaling and serine phosphorylation of IRS‐1 in muscle cells/skeletal muscle are temporal and reversible. In addition, in both lean and obese rats, leucine‐induced impairment of whole body insulin sensitivity of glucose and amino acid is reversible within 24 h of treatment. Our data suggest that the use of leucine/amino acid in body weight management is unlikely to carry any lasting negative effect on insulin sensitivity.

## Introduction

High‐protein diets (≥25% energy intake as proteins) (Eisenstein et al. 2002) have been used in the management of obesity. The mechanisms of action of these diets include increased thermogenesis, enhancement of satiety (Westerterp‐Plantenga et al. 1999), and increased loss of body fat mass (Layman et al. 2005). Amino acids (AAs) from dietary proteins not only serve as substrates for body protein synthesis but can also activate the mammalian (mechanistic) target of rapamycin mTOR complex 1 (mTORC1) signaling, a pathway that is essential in regulating muscle mass (reviewed in Adegoke et al. 2012; Efeyan et al., 2012a,b). In this regard, the most well‐characterized substrates of mTORC1 are ribosomal protein S6 kinase 1 (S6K1) and eukaryotic initiation factor 4E (eIF4E)‐binding proteins (4E‐BPs) (Dowling et al. 2010; Thoreen et al. 2012).

At odds with the benefits attributed to high‐protein diets are data that implicate them in the pathogenesis of insulin resistance, a metabolic derangement that can lead to type 2 diabetes, cardiovascular disease, and some cancers. Some studies have shown a link between high‐protein diets and glucose intolerance, increased gluconeogenesis, insulin resistance, and type 2 diabetes (Lariviere et al. 1994; Linn et al. 2000; Schulze et al. 2003; Song et al. 2004). Amino acids, particularly leucine and other branched chain amino acids (BCAA), which are potent activators of mTORC1, have been identified as major players in this (Krebs et al. 2002; Newgard et al. 2009; Adams 2011). In addition to stimulating muscle anabolism, activated mTORC1/S6K1 can phosphorylate insulin receptor substrate 1 (IRS‐1) on serine residues, which can lead to insulin resistance (Tremblay et al. 2007).

A feature of many signaling pathways is the reversibility of the signaling cascade. For example, following the initiation of insulin signaling, protein tyrosine phosphatase 1B (PTB1B), suppressor of cytokine signaling 1 (SOCS1) and SOCS3 are activated, leading to reduced phosphorylation of IRS1 (Shepherd 2005). In addition, activation of phosphate and tensin homolog on chromosome‐10 (PTEN) converts phosphatidylinositol 3,4,5‐triphosphate (PIP3) to PIP2 (Song et al. 2012), leading to the suppression of AKT signaling. Although deregulation of these phosphatases may lead to insulin resistance, their functions are a part of reversible switches critical for normal insulin action (Jin and Pawson 2012). It is likely that amino acid‐induced effect of mTORC1/S6K1 in phosphorylating IRS‐1 and the attendant insulin resistance are normal but reversible physiological events, at least in healthy individuals. However, the dynamics of mTORC1/S6K1 effect on IRS‐1 serine phosphorylation and insulin resistance remains to be examined. Here, our objectives were to (1) examine the time course of the effect of leucine‐enriched AA medium and/or insulin on mTORC1 signaling and the associated IRS‐1 serine phosphorylation and (2) examine the dynamics of the effect of this AA mixture or leucine on glucose transport in myotubes and on whole body insulin resistance of glucose and amino acid metabolism in healthy and insulin‐resistant rats.

## Materials and Methods

### Reagents

Fetal bovine serum and antibiotic–antimycotic preparations were purchased from Life Technologies (Burlington, Ontario, Canada). Immobilon Western Horseradish Peroxidase (HRP) chemiluminescence substrate was obtained from Millipore Corporation (Billerica, MA); l‐leucine, phosphatase and protease inhibitor cocktails from Sigma‐Aldrich (St. Louis, MO); *α*‐Eagle's minimal essential medium (AMEM) and trypsin from Wisent (St Bruno, Quebec, Canada); and l‐leucine dehydrogenase from Calbiochem (EMD Millipore). Amino acid‐free medium (RPMI 1640) was obtained from US Biological (Swampscott, MA). Glucose solutions and glucose/lactate buffers were obtained from Interscience (Markham, ON Canada).

### Antibodies

Antibodies to phospho (ph)‐S6K1 (T389), total S6K1, ph‐IRS‐1 (S307/612, S636/639), total IRS‐1, ph‐Akt (T308), and HRP‐conjugated secondary antibodies (anti‐rabbit and anti‐mouse) were purchased from Cell Signaling Technology (Danvers, MA). Antibodies to *γ* tubulin were obtained from Sigma‐Aldrich.

### Amino acid and insulin activation of mTORC1/S6K1 in myotubes

**Figure 1.**
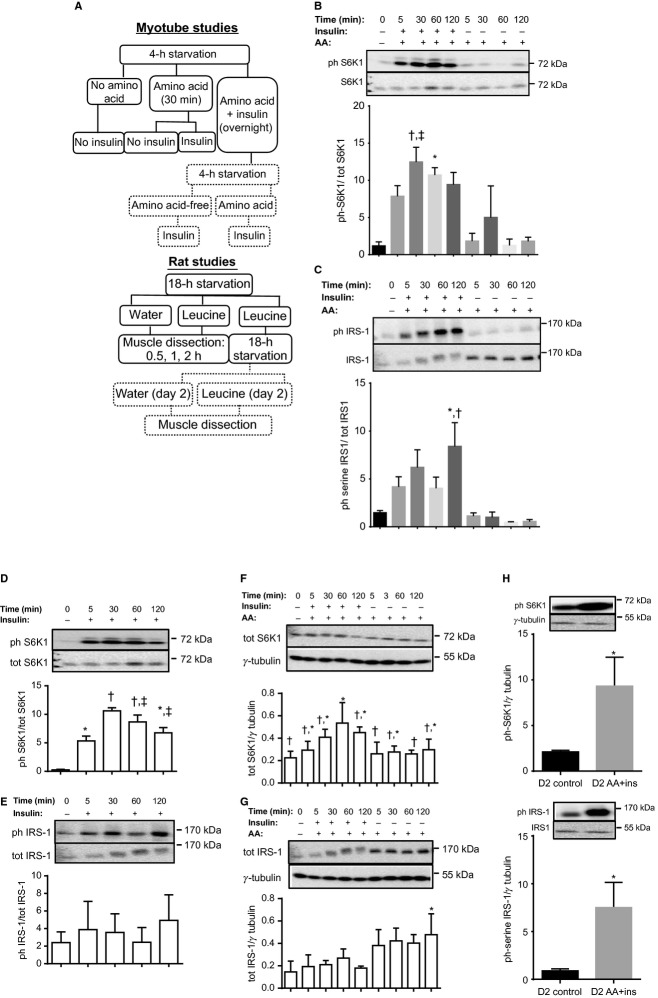
In L6 myotubes, amino acid and insulin induce S6K1 (T389) and IRS‐1 (serine residues) phosphorylation in a reversible manner. (A) Experimental design for myotube (top) and rat (bottom) studies. Studies conducted on day 2 are in dotted boxes. (B, C) Myotubes were starved for 4 h and then incubated for 30 min in either an amino acid‐free solution or AMEM with added leucine to a final concentration of 800 *μ*mol/L (AA). Incubation with AA continued for up to 2 h in the presence or absence of insulin. Cells were harvested and lysates probed for phosphorylated (B) S6K1 (T389) and (C) IRS‐1 (S 307/612/636/639). Data are mean ± SD. In (B), *P <**0.05 versus time 0, AA at 60 and 120 min; ^†^*P *<**0.01 versus time 0, AA at 60 and 120 min; ^‡^*P *<**0.05 versus AA at 5 min. In (C), *P <**0.05 versus time 0; ^†^*P *<**0.01 versus AA at all time points. (D, E) Phosphorylated and total S6K1 and IRS‐1 in myotubes incubated with insulin for different lengths of time. Data are mean ± SD. Bars with different symbols differ from one another (*P *<**0.05). (F, G) Total S6K1 and IRS‐1 in myotubes incubated with leucine‐enriched amino acid (AA) with or without insulin for different lengths of time. Data are mean ± SD. In (F), bars with different symbols differ from one another (*P *<**0.05). In (G), *P <**0.05 compared to time 0. (H) Myotubes treated as in (B) were incubated in AA and insulin overnight. They were then starved for 4 h (D2 control) and subjected to AA and insulin stimulation (D2 AA+ins). Cell lysates were probed for phosphorylated S6K1 and IRS‐1. Data are mean ± SD, *P ≤**0.05 versus D2 control.

L6 myotubes were starved for 4 h in serum‐free AMEM, following which cells were incubated in either serum‐ and AA‐free medium or leucine‐enriched AMEM (final concentration of leucine: 800 *μ*mol/L), for 30 min (Fig. 1A, top). We used a leucine‐enriched medium because of the demonstrated effects of this amino acid on mTORC1 signaling (Newgard 2012). After this, incubation with the AA mixture continued in either the presence or the absence of 100 nmol/L insulin for between 5 min and 2 h. Cells were then harvested in a lysis buffer (25 mmol/L Tris, pH 7.5, 2% SDS, 1 mmol/L EDTA, 1 mmol/L DTT, supplemented with protease and phosphatase inhibitor cocktails to 10 *μ*L/mL). In another experiment, myotubes were incubated in AA and insulin overnight. The next day, and to simulate a washout, the cells were starved for 4 h in serum‐free AMEM and then incubated in either AA or an AA‐free medium for 30 min, followed by incubation in insulin for an additional 30 min. Cells were then harvested and immunoblot analysis for the indicated antigens was carried out. In other experiments, after the incubation in AA and/insulin as described above, insulin‐stimulated glucose transport was conducted (Pimenta et al. 2008).

### Rat studies

Young male Sprague–Dawley rats (50–70 g) were purchased from Charles River Laboratories Inc. (Quebec, Canada). They were left to acclimatize for 1 week in the animal care facility at York University while being maintained at the standard 12:12‐h light–dark cycle at 22–23°C. They had free access to rat chow (product #D12450B, Research Diets, New Brunswick, NJ) and water. After acclimatization, the animals were handled 2–3 times per week in order to reduce stress due to handling on the day of the experiment. All experiments were approved by the York University Animal Care Committee and were performed in accordance with the Canadian Council for Animal Care guidelines.

Rats were food deprived for 18 h but had access to water. They were then divided into three groups (Fig. 1A, bottom). The first group (control [stv]) was gavaged with double distilled water at a dose of 2.4 mL/100 g body weight. The second group was gavaged with leucine (1 g/50 mL ddH_2_O, 2.4 mL/100 g body weight). This amount is equivalent to 0.48 g leucine/kg, and represents ~40% of a rat's daily leucine consumption (Serino et al. 2011). Rats were sacrificed at different times (0.5–2 h) after gavage and soleus muscle dissected. The third group was gavaged with leucine and, 2 h later, returned to food. This group was then again food‐deprived overnight and divided into two subgroups. One subgroup was regavaged on day 2 with water while the other was gavaged with leucine. Rats were sacrificed at either 0.5 or 1 h postgavage and soleus muscle dissected. To examine whether the dynamics of regulation of S6K1 and IRS1 might be different in insulin‐resistant state, identical experiments were conducted in rats fed high‐fat diet (60% kcal as fat, product # D2492, Research Diets) for 8 weeks to render them insulin resistant.

### Insulin tolerance test

Following a 6 h food deprivation, rats were gavaged with either water or leucine. Thirty minutes following gavage, blood samples were taken via tail nick and then a subcutaneous insulin (Humulin R, Eli Lilly Canada Inc., Toronto, Ontario, Canada) injection was administered (0.75 U/kg body weight). Blood samples were collected at various times postinjection, and the resulting plasma analyzed for glucose (YSI Glucose/Lactate analyzer), total BCAA (Chevalier et al. 2004) concentrations, and for insulin using a commercial kit (Crystal Chem, Downers Grove, IL).

### Western blot

Soleus muscle samples were homogenized as described (Zargar et al. 2011). Equal amount of proteins in the supernatant fraction was separated by SDS‐PAGE. Phosphorylated S6K1 (T389) and IRS‐1 (S307/612/636/639) were detected by immunoblotting (Zargar et al. 2011).

### Statistical analysis

Data from cell culture studies were analyzed using a one‐way ANOVA and Bonferroni post hoc test (GraphPad Prism 6.00, GraphPad Software Inc., La Jolla, CA). For rat studies, S6K1 and IRS‐1 data were analyzed by one‐way ANOVA, whereas glucose, BCAA, and insulin were analyzed by two‐way ANOVA and Tukey's multiple comparison test. Statistical significance was set at *P *<**0.05.

## Results

### Amino acid‐ and insulin‐induced mTORC1 activation and IRS‐1 serine phosphorylation in myotubes are reversible

Incubation of L6 myotubes with a combination of AA and insulin increased T389 S6K1 (Fig. 1B) and, in a time‐dependent manner, serine phosphorylation of IRS‐1 (Fig. 1C). Under the conditions tested, AA alone had no effect. Insulin by itself stimulated S6K1 phosphorylation (Fig. 1D) but had no significant effect on IRS‐1 serine phosphorylation (Fig. 1E). In addition, incubation with AA plus insulin increased S6K1 abundance at the 1 h time point (Fig. 1F) but no significant effect was seen on total IRS‐1 until 2 h when the abundance of the protein was increased (Fig. 1G).

To examine the dynamics of these modifications, we incubated myotubes overnight in a medium that contained both AA and insulin. Following this, the starvation–refeeding experiment was then repeated. As seen in Figure 1H, T389 S6K1 and IRS‐1 serine phosphorylation was low in starved cells, even though these cells had been incubated in AA plus insulin overnight. When restimulated, however, phosphorylation of these proteins was induced. Therefore, in L6 myotubes, the effect of AA and insulin in activating S6K1 and inducing serine phosphorylation of IRS‐1 is reversible.

### In myotubes, the inhibitory effect of AA on insulin‐stimulated glucose transport is reversible

**Figure 2.**
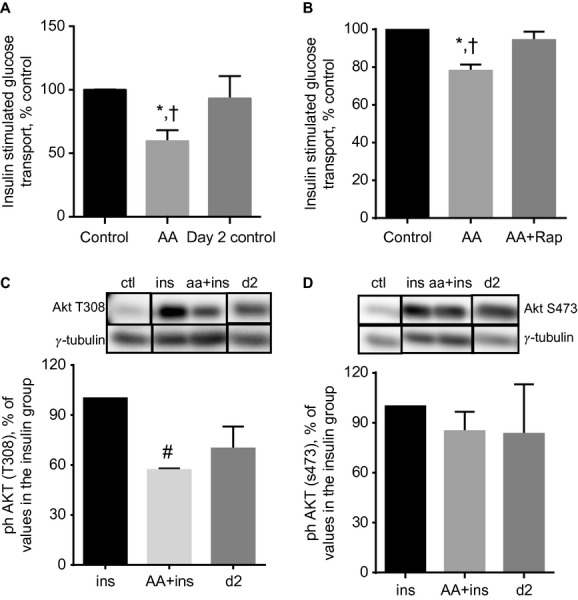
Impairment of insulin‐stimulated glucose transport in myotubes treated with AA + insulin is reversible. (A) Myotubes were starved for 4 h and then incubated for 30 min in either an amino acid‐free solution (Control) or leucine‐enriched AA mixture (AA). Following this, insulin‐stimulated glucose transport was measured. In another group, incubation in AA and insulin continued overnight. Cells were then starved (day 2 control) and insulin‐stimulated glucose transport was then measured. Data are mean ± SE of 5–6 independent experiments; ^†^*P *<**0.01 versus Control; *P <**0.05 versus Day 2 control. (B) Treatments as in (A) except that incubation with AA was carried out with coincubation with DMSO (AA) or 50 nmol/L rapamycin (AA + Rap). ^†^*P *<**0.01 versus Control; *P <**0.05 versus AA + Rap. (C, D). Myotubes were starved for 4 h and then incubated with or without AA. Cells were then incubated with insulin in the absence (ins) or presence of AA (aa + ins). Control (ctl) group was not treated with insulin or AA. A fourth group was treated with AA + insulin overnight. Cells were then starved for 4 h and stimulated with insulin (d2). AKT phosphorylation was then analyzed. In the bottom panels, data are expressed as a % of the ins group. Mean ± SE of three experiments (AKT [S473]) or mean ± SD of two experiments (AKT [T308]); ^#^*P *<**0.05 versus ins.

Activation of mTORC1/S6K1 and serine phosphorylation of IRS‐1 have been linked to insulin resistance of glucose transport. We therefore examined whether the dynamics we observed in the regulation of these proteins also applied to their effects on glucose transport. AA suppressed insulin‐stimulated glucose transport by 40% (*P *<**0.01, Fig. 2A). Next, some of the myotubes were incubated in AA and insulin overnight, and then starved the next day for 4 h. Insulin‐stimulated glucose transport in these cells was not different from that observed in the control group (Fig. 2A). These results suggest that the impairment of insulin‐stimulated glucose uptake in L6 myotubes is a temporal and reversible phenomenon. Finally, the suppressive effect of AA and insulin on glucose transport is mTORC1 dependent as incubation with rapamycin, an mTORC1 inhibitor, abolished the effect (Fig. 2B).

Because AKT is implicated in insulin‐stimulated glucose transport, we examined the regulation of this protein. AKT T308 and S473 phosphorylation was induced by insulin (Fig. 2C and D). The effect of insulin on T308 phosphorylation was partially reversibly modified in the presence of AA.

### In lean rats, leucine gavage reversibly increases soleus muscle phosphorylation of S6K1 and IRS‐1 and suppresses whole body insulin sensitivity of glucose

**Figure 3.**
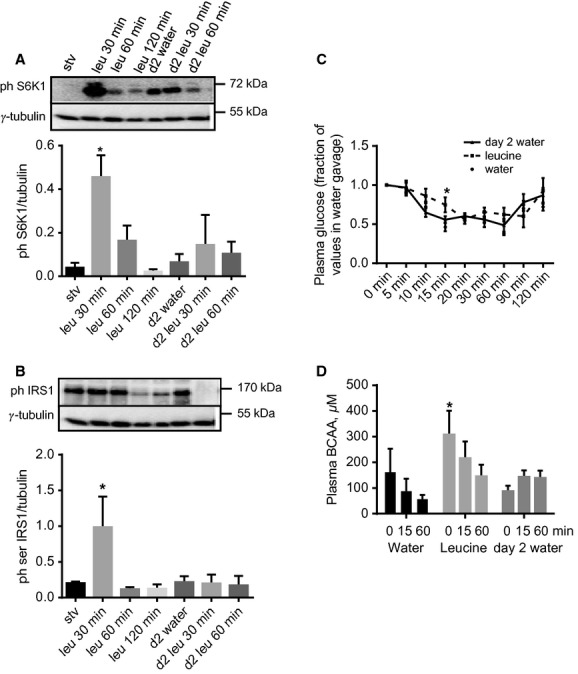
In lean rats, leucine gavage reversibly increases soleus muscle phosphorylation of S6K1 and IRS‐1 (serine residues) and whole body insulin sensitivity. Rats were starved for 18 h and then gavaged with either water (stv) or leucine. They were sacrificed at different times following gavage. Soleus muscles homogenates were analyzed for (A) phospho S6K1 and (B) serine phosphorylated IRS‐1. Another group of rats was gavaged with leucine and 2 h later returned to food. Rats were then starved overnight and, the following day, were gavaged with either water (d2 water) or leucine (the 2 bars to the right in A and B). They were sacrificed at the times indicated and soleus muscles were analyzed for phospho S6K1 and IRS‐1. Mean ± SE,* n *=**5–7, *P <**0.05 versus stv. (C) Plasma glucose concentrations, as fractions of time zero value, in rats that were gavaged with water or leucine, and then subjected to ITT 30 min after gavage. A third group was treated as described in (B) and the following day was gavaged with water, followed by ITT (day 2 water). Mean ± SD,* n *=**4–7, *P <**0.05 versus water (i.e., rats gavaged with water on day 1). (D) Plasma BCAA during the ITT described in (C). Mean ± SD,* n *=**4–7, *P <**0.05, versus T0 in day 2 water group.

To test whether these results could be mimicked in vivo and in line with the significance of branched chain AA in promoting insulin resistance (Newgard et al. 2009), we compared the effect of leucine or water gavage in young healthy male rats that had been food deprived for 18 h. Thirty minutes after gavage, leucine, but not water, significantly increased S6K1 T389 (~12‐fold, *P *<**0.001) and IRS‐1 serine phosphorylation (~5‐fold, *P *<**0.05, Fig. 3A and B) in soleus muscle. A group of rats that had been gavaged with leucine was returned to food 2 h after gavage, and then food‐deprived overnight. They were then regavaged with either water or leucine and sacrificed at various time points. Following overnight food deprivation after the initial leucine gavage, S6K1 and IRS‐1 phosphorylation was reduced to basal in rats gavaged with water (referred to as “d2 water” in Fig. 3A and B).

To determine the functional consequences of these results, another group of rats was food deprived for 6 h and then gavaged with either water or leucine. Thirty minutes later, an insulin tolerance test (ITT) was conducted. Rats gavaged with leucine had significantly higher plasma blood glucose concentration only at 15 min from the initiation of ITT (Fig. 3C; mmol/L ± SE values at T0 and T15: Water gavage: 8.2 ± 0.2 vs. 3.9 ± 0.4; Leucine gavage: 7.4 ± 1 vs. 5.2 ± 0.2). A third group of rats was gavaged with leucine, starved overnight, and regavaged the next day with water before being administered the ITT. Blood glucose concentrations were not different from the values in the group gavaged with water on day 1 (Fig. 3C).

In young animals, insulin stimulates amino acid utilization for protein synthesis and suppresses proteolysis, leading to a decrease in plasma amino acid concentrations, especially those of the BCAA (Garlick and Grant 1988; Luzi et al. 1996). To obtain a measure of the effect of leucine gavage on whole body protein metabolism, we determined plasma total BCAA concentrations during the ITT. In response to insulin, plasma BCAA was unchanged in rats that were gavaged with water. In those that received leucine gavage, insulin administration rapidly suppressed plasma BCAA levels and the levels remained low in rats that were studied on day 2 (Fig. 3D).

### In insulin‐resistant rats, the effects of leucine on whole body glucose and amino acid metabolism are reversible

**Figure 4.**
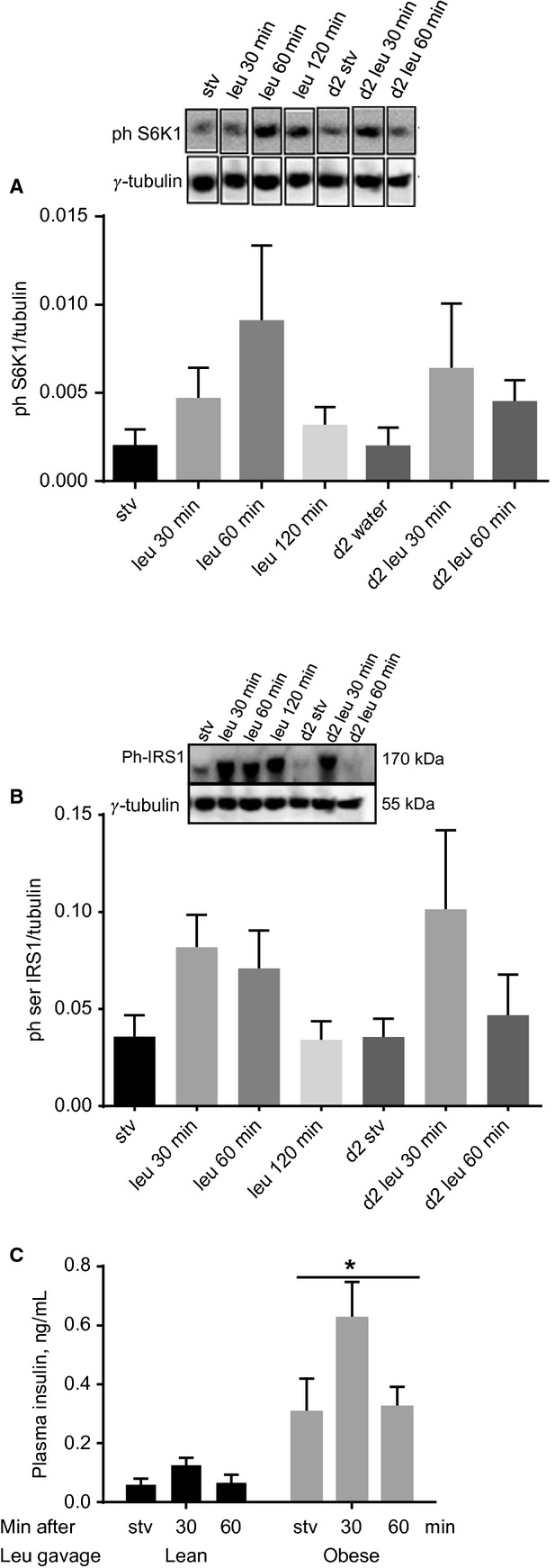
In obese rats, leucine gavage has no effect on soleus muscle phosphorylation of S6K1 and IRS‐1. Rats made obese by being fed high‐fat diet were gavaged with water or with leucine as described in Figure 3. Soleus muscle homogenates were analyzed for (A) phospho S6K1 and (B) serine phosphorylated IRS‐1. Another group of rats was gavaged with leucine and 2 h later returned to food. The rats were then starved overnight and the following day regavaged with either water or leucine (last three columns to the right in A and B). Mean ± SE;* n *=**6. (C) Plasma insulin in lean or obese rats gavaged with leucine. Mean ± SE;* n *=**6, *P <**0.001 versus lean.

There is an impairment of insulin signaling in skeletal muscle of obese insulin‐resistant individuals. Since obese individuals frequently attempt to lose weight by using a high‐protein diet, it was necessary to examine whether the pattern of response to leucine gavage we saw in lean animals was preserved in rats made obese/insulin resistant by high‐fat diet feeding. S6K1 and IRS‐1 (serine) phosphorylation was detected even in soleus muscles of fasted obese rats. Although these measures increased in response to leucine gavage, the values were not significantly different from basal (Fig. 4A and B). As was done for lean rats, we also studied on day 2 rats that had been gavaged with leucine on day 1. As seen in animals studied on day 1, the effect of leucine gavage on muscle phosphorylation of S6K1 was not significant (Fig. 4A and B).

Although plasma insulin was higher in obese relative to lean rats, leucine gavage had no significant effect on this hormone (Fig. 4C).

**Figure 5.**
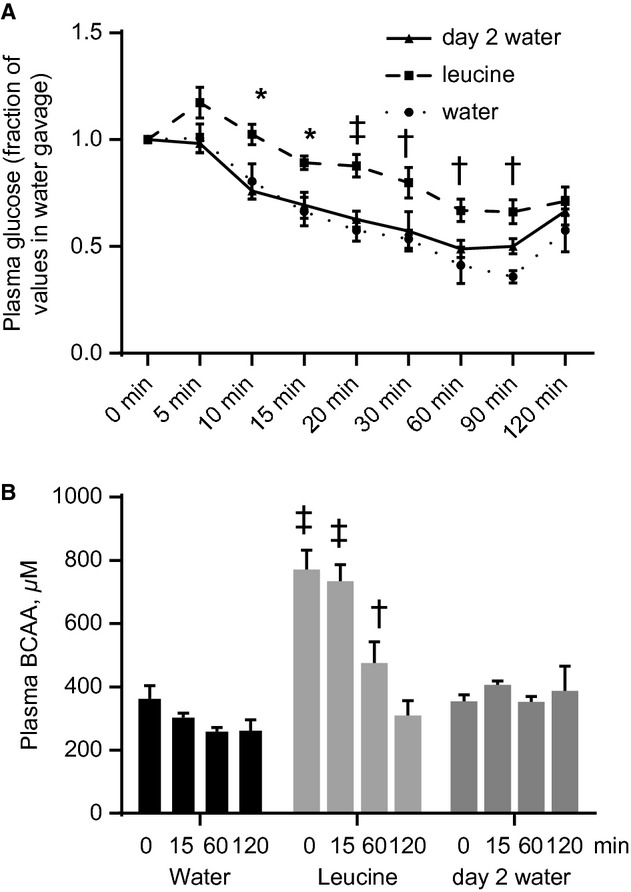
Leucine gavage reversibly modifies whole body insulin sensitivity of glucose and amino acid metabolism in obese rats. (A) Plasma glucose concentrations, as fractions of time zero value, in obese rats that were gavaged with water or leucine, and then subjected to ITT 30 min after gavage, as described in Figure 3. A third group was treated as described in Figure 3C and the following day was gavaged with water, followed by ITT (day 2 water). (B) Plasma BCAA during the ITT. Mean ± SE,* n *=**6–7. *P <**0.05, ^†^*P *<**0.01, ^‡^*P *<**0.001 versus corresponding water at the indicated times.

Fifteen minutes from the commencement of ITT in lean rats gavaged with water, plasma glucose was <50% of time zero value (Fig. 3C). At equivalent time in insulin‐resistant rats gavaged with water, the effect of insulin on blood glucose concentration was not as marked (mmol/L ± SE at T0 and T15: 8.8 ± 0.2 and 5.8 ± 0.5, compare Fig. 5A to Fig. 3C), confirming the efficacy of the high‐fat diet in inducing insulin resistance. In obese rats gavaged with leucine, compared to those that received water, plasma glucose concentration was significantly higher at all time points between 10 and 90 min (Fig. 5A). A group of rats was gavaged with leucine and then given access to food 2 h after the gavage. When this group was food‐deprived overnight and studied the following day, plasma blood glucose concentrations during the ITT were not different from the values in the group that was gavaged with water and studied on day 1 (Fig. 5A).

Plasma BCAA concentrations were higher in obese rats; in fact, the values in those gavaged with water were similar to the peak level seen in lean animals that were gavaged with leucine (compare Figs. 3D and 5B). Contrary to the observations in lean rats, plasma BCAA levels did not return to basal until 2 h from the start of ITT, consistent with insulin resistance of whole body protein metabolism. When rats gavaged with leucine were studied on day 2, BCAA levels remained at the basal level.

## Discussion

Insulin resistance nucleates a number of metabolic diseases, including type 2 diabetes and cardiovascular disease (DeFronzo 2010), nonalcoholic liver disease (Bugianesi et al. 2010), and some cancers (Tsugane and Inoue 2010). Therefore, interventions that improve insulin sensitivity hold promise for the prevention and/or management of those conditions. Hence, the possibility that a treatment used in body weight management (high‐protein diet) could induce insulin resistance has serious clinical implications. Using in vitro and in vivo approaches, we demonstrated that AA plus insulin (myotube studies) or leucine (rat studies) reversibly modified S6K1 T389 and IRS‐1 serine phosphorylation in muscle cells and skeletal muscle, and caused temporal and reversible insulin resistance of glucose and amino acid metabolism in both lean and obese rats.

Although the possible roles of AA in regulating insulin sensitivity have been proposed since the 1960s (Felig et al. 1969, 1970), it is only in the last few years that the putative mechanisms that underlie this phenomenon have been studied (Newgard 2012). Whereas, earlier studies implicated amino acids and high‐protein diets as mediators of glucose intolerance, hyperinsulinemia, and insulin resistance (Lariviere et al. 1994; Linn et al. 2000; Schulze et al. 2003; Song et al. 2004), others have shown beneficial effects of these diets. These include enhancement of insulin sensitivity in the elderly (Solerte et al. 2008) and in obese mice (Macotela et al. 2011), and beneficial effects against cardiac and skeletal muscle mitochondrial defects in old rats (D'Antona et al. 2010). A study with >4000 participants of different ethnicities showed that higher consumption of BCAA is associated with lower prevalence of overweight/obesity (Qin et al. 2011).

While other mechanisms through which AA may induce insulin resistance are possible, it is the one mediated by mTORC1/S6K1 that has been intensively studied. Activated S6K1 induces phosphorylation of IRS‐1 on multiple serine residues, a modification that not only impedes tyrosine phosphorylation of IRS‐1 but is also thought to stimulate its degradation. These effects on IRS1 lead to a quenching of insulin signaling (Tremblay et al. 2007). Indeed mice that lack S6K1 are protected against diet‐induced insulin resistance (Um et al. 2004). Our data demonstrating that rapamycin blocked the effect of AA on glucose transport in myotubes and rat data showing increased S6K1 activation and IRS‐1 serine phosphorylation along with insulin resistance of glucose metabolism in response to leucine gavage are consistent with this.

Majority of the studies on insulin resistance typically focus on glucose metabolism, even though insulin also regulates protein synthesis and/or breakdown (Marliss and Gougeon 2002), especially in skeletal muscle of rodent (Wang et al. 2006; Kimball and Jefferson 2010) and human whole body studies (Halvatsiotis et al. 2002; Pereira et al. 2008). In fact, there is some evidence to link impaired insulin regulation of protein metabolism, which would lead to increased availability of free (glucogenic) AA, to the increased gluconeogenesis observed in insulin‐resistant individuals (Chevalier et al. 2006). Our data on plasma BCAA showed that when leucine gavage is administered prior to ITT, the elevated plasma BCAA is rapidly brought back to normal, consistent with the idea that in lean rats, these amino acids do not impair insulin sensitivity, as others have shown (Bassil et al. 2011). Obviously this measure reflects whole body average and does not allow identification of tissue‐specific effect. However, because increased AA disposal via elevated muscle protein synthesis is a main effect of insulin (Davis and Reeds 1998; Chevalier et al. 2004), it is likely that the disappearance of AA is linked to insulin‐induced stimulation of muscle protein synthesis. The picture was quite different in obese insulin‐resistant animals. Plasma BCAA was already elevated in these animals, as has been reported by others (Newgard et al. 2009; Wang et al. 2011). During the ITT, the normalization of these AA took markedly longer (15 min in lean vs. >60 min in obese). Moreover, the reduction in BCAA in response to insulin was only to the pre‐ITT level, but not to the level seen in lean rats. In spite of this apparent impairment of whole body insulin sensitivity of protein metabolism, the alteration is nevertheless reversible. Therefore, leucine administration, and likely acute dietary protein consumption, in obese individuals are unlikely to worsen the preexisting insulin resistance.

At least two mechanisms may explain the reversibility of mTORC1/S6K1 signaling and IRS‐1 serine phosphorylation. Specific phosphatases may dephosphorylate these kinases. For example, protein tyrosine phosphatase‐1B (PTP1B) can dephosphorylate IRS1 (Lazar and Saltiel 2006) while *PH* domain *l*eucine‐rich repeat *p*rotein *p*hosphatase (PHLPP) (Liu et al. 2011) and B’ regulatory subunit of protein phosphatase 2A (PP2A‐B’) (Hahn et al. 2010) have been shown to dephosphorylate S6K1. Compared to the kinases that phosphorylate these proteins, the phosphatases tend to have broader specificities and mechanisms of their regulation are relatively poorly understood. A second mechanism of the reversibility of signaling to these proteins is targeted proteolysis of phosphorylated S6K1 and IRS‐1. Ubiquitin protein ligases that target mTOR (Mao et al. 2008) and IRS‐1 (Rui et al. 2002) for degradation by the proteasome have been described, and there is evidence that S6K1 too may be degraded by the proteasome (Gao et al. 2009). It will be interesting to examine whether entities that target them for degradation are regulated by AA in a manner that can explain the observed reversible phosphorylation of S6K1 and IRS‐1 in response to leucine.

In summary, we provide evidence that AA/leucine‐induced impairment of skeletal muscle insulin signaling and whole body insulin sensitivity of glucose in healthy rat is modest and reversible. In obese rats, although leucine administration further impaired insulin regulation of whole body glucose and amino acid metabolism, the effects were reversible. In studies that have attributed insulin resistance to high‐protein diet, leucine is thought to be the main mediator of this effect, therefore, our data have implications for human nutrition and management of metabolic diseases. While these data need to be confirmed in studies that examine the effects of long‐term high dietary protein intakes, our results suggest that the benefits derivable from the use of high‐protein diets by obese individuals are unlikely to be associated with worsening of insulin sensitivity.

## Acknowledgments

We thank Rolando Ceddia for guidance on glucose transport in muscle cells.

## Conflict of Interest

None declared.

## References

[b1] Adams S. H. 2011 Emerging perspectives on essential amino acid metabolism in obesity and the insulin‐resistant state. Adv. Nut.: Int. Rev. J.; 2:445-456. 10.3945/an.111.000737 PMC3226382 22332087

[b2] Adegoke O. A. Abdullahi A. Tavajohi‐Fini P. 2012 mTORC1 and the regulation of skeletal muscle anabolism and mass. Appl. Physiol. Nutr. Metab.; 37:395-406. 22509811 10.1139/h2012-009

[b3] Bassil M. Burgos S. Marliss E. B. Morais J. A. Chevalier S. Gougeon R. 2011 Hyperaminoacidaemia at postprandial levels does not modulate glucose metabolism in type 2 diabetes mellitus. Diabetologia; 54:1810-1818. 21437771 10.1007/s00125-011-2115-7

[b4] Bugianesi E. Moscatiello S. Ciaravella M. F. Marchesini G. 2010 Insulin resistance in nonalcoholic fatty liver disease. Curr. Pharm. Des.; 16:1941-1951. 20370677 10.2174/138161210791208875

[b5] Chevalier S. Gougeon R. Kreisman S. H. Cassis C. Morais J. A. 2004 The hyperinsulinemic amino acid clamp increases whole‐body protein synthesis in young subjects. Metabolism; 53:388-396. 15015153 10.1016/j.metabol.2003.09.016

[b6] Chevalier S. Burgess S. C. Malloy C. R. Gougeon R. Marliss E. B. Morais J. A. 2006 The greater contribution of gluconeogenesis to glucose production in obesity is related to increased whole‐body protein catabolism. Diabetes; 55:675-681. 16505230 10.2337/diabetes.55.03.06.db05-1117

[b7] D'Antona G. Ragni M. Cardile A. Tedesco L. Dossena M. Bruttini F. 2010 Branched‐chain amino acid supplementation promotes survival and supports cardiac and skeletal muscle mitochondrial biogenesis in middle‐aged mice. Cell Metab.; 12:362-372. 20889128 10.1016/j.cmet.2010.08.016

[b8] Davis T. A. Reeds P. J. 1998 The roles of nutrition, development and hormone sensitivity in the regulation of protein metabolism: an overview. J. Nutr.; 128:340S-341S. 9478020 10.1093/jn/128.2.340S

[b9] DeFronzo R. A. 2010 Insulin resistance, lipotoxicity, type 2 diabetes and atherosclerosis: the missing links. The Claude Bernard Lecture 2009. Diabetologia; 53:1270-1287. 20361178 10.1007/s00125-010-1684-1 PMC2877338

[b10] Dowling R. J. Topisirovic I. Alain T. Bidinosti M. Fonseca B. D. Petroulakis E. 2010 mTORC1‐mediated cell proliferation, but not cell growth, controlled by the 4E‐BPs. Science; 328:1172-1176. 20508131 10.1126/science.1187532 PMC2893390

[b11] Efeyan A. Zoncu R. Chang S. Gumper I. Snitkin H. Wolfson R. L. 2012a Regulation of mTORC1 by the Rag GTPases is necessary for neonatal autophagy and survival. Nature; 493:679-683. 23263183 10.1038/nature11745 PMC4000705

[b12] Efeyan A. Zoncu R. Sabatini D. M. 2012b Amino acids and mTORC1: from lysosomes to disease. Trends Mol. Med.; 18:524-533. 22749019 10.1016/j.molmed.2012.05.007 PMC3432651

[b13] Eisenstein J. Roberts S. B. Dallal G. Saltzman E. 2002 High‐protein weight‐loss diets: are they safe and do they work? A review of the experimental and epidemiologic data. Nutr. Rev.; 60:189-200. 12144197 10.1301/00296640260184264

[b14] Felig P. Marliss E. Cahill G. F. Jr 1969 Plasma amino acid levels and insulin secretion in obesity. N. Engl. J. Med.; 281:811-816. 5809519 10.1056/NEJM196910092811503

[b15] Felig P. Marliss E. Cahill G. F. Jr 1970 Are plasma amino acid levels elevated in obesity? N. Engl. J. Med.; 282:166 5409545 10.1056/nejm197001152820315

[b16] Gao Z. Yin J. Zhang J. He Q. McGuinness O. P. Ye J. 2009 Inactivation of NF‐kappaB p50 leads to insulin sensitization in liver through post‐translational inhibition of p70S6K. J. Biol. Chem.; 284:18368-18376. 19433583 10.1074/jbc.M109.007260 PMC2709339

[b17] Garlick P. J. Grant I. 1988 Amino acid infusion increases the sensitivity of muscle protein synthesis in vivo to insulin. Effect of branched‐chain amino acids. Biochem. J.; 254:579-584. 3052439 10.1042/bj2540579 PMC1135117

[b18] Hahn K. Miranda M. Francis V. A. Vendrell J. Zorzano A. Teleman A. A. 2010 PP2A regulatory subunit PP2A‐B’ counteracts S6K phosphorylation. Cell Metab.; 11:438-444. 20444422 10.1016/j.cmet.2010.03.015

[b19] Halvatsiotis P. Short K. R. Bigelow M. Nair K. S. 2002 Synthesis rate of muscle proteins, muscle functions, and amino acid kinetics in type 2 diabetes. Diabetes; 51:2395-2404. 12145150 10.2337/diabetes.51.8.2395

[b20] Jin J. Pawson T. 2012 Modular evolution of phosphorylation‐based signalling systems. Philos. Trans. Roy. Soc. B: Biol. Sci.; 367:2540-2555. 10.1098/rstb.2012.0106 PMC3415845 22889906

[b21] Kimball S. R. Jefferson L. S. 2010 Control of translation initiation through integration of signals generated by hormones, nutrients, and exercise. J. Biol. Chem.; 285:29027-29032. 20576612 10.1074/jbc.R110.137208 PMC2937931

[b22] Krebs M. Krssak M. Bernroider E. Anderwald C. Brehm A. Meyerspeer M. 2002 Mechanism of amino acid‐induced skeletal muscle insulin resistance in humans. Diabetes; 51:599-605. 11872656 10.2337/diabetes.51.3.599

[b23] Lariviere F. Chiasson J. L. Schiffrin A. Taveroff A. Hoffer L. J. 1994 Effects of dietary protein restriction on glucose and insulin metabolism in normal and diabetic humans. Metabolism; 43:462-467. 8159104 10.1016/0026-0495(94)90077-9

[b24] Layman D. K. Evans E. Baum J. I. Seyler J. Erickson D. J. Boileau R. A. 2005 Dietary protein and exercise have additive effects on body composition during weight loss in adult women. J. Nutr.; 135:1903-1910. 16046715 10.1093/jn/135.8.1903

[b25] Lazar D. F. Saltiel A. R. 2006 Lipid phosphatases as drug discovery targets for type 2 diabetes. Nat. Rev. Drug Discov.; 5:333-342. 16582877 10.1038/nrd2007

[b26] Linn T. Santosa B. Gronemeyer D. Aygen S. Scholz N. Busch M. 2000 Effect of long‐term dietary protein intake on glucose metabolism in humans. Diabetologia; 43:1257-1265. 11079744 10.1007/s001250051521

[b27] Liu J. Stevens P. D. Li X. Schmidt M. D. Gao T. 2011 PHLPP‐mediated dephosphorylation of S6K1 inhibits protein translation and cell growth. Mol. Cell. Biol.; 31:4917-4927. 21986499 10.1128/MCB.05799-11 PMC3233022

[b28] Luzi L. Castellino P. DeFronzo R. A. 1996 Insulin and hyperaminoacidemia regulate by a different mechanism leucine turnover and oxidation in obesity. Am. J. Physiol. Endocrinol. Metab.; 270:E273-E281. 10.1152/ajpendo.1996.270.2.E273 8779949

[b29] Macotela Y. Emanuelli B. Bang A. M. Espinoza D. O. Boucher J. Beebe K. 2011 Dietary leucine–an environmental modifier of insulin resistance acting on multiple levels of metabolism. PLoS One; 6:e21187 21731668 10.1371/journal.pone.0021187 PMC3120846

[b30] Mao J. H. Kim I. J. Wu D. Climent J. Kang H. C. DelRosario R. 2008 FBXW7 targets mTOR for degradation and cooperates with PTEN in tumor suppression. Science; 321:1499-1502. 18787170 10.1126/science.1162981 PMC2849753

[b31] Marliss E. B. Gougeon R. J. 2002 Diabetes mellitus, Lipidus Etâ€¦ Proteinus!. Diabetes Care; 25:1474-1476. 12145253 10.2337/diacare.25.8.1474

[b32] Newgard C. B. 2012 Interplay between lipids and branched‐chain amino acids in development of insulin resistance. Cell Metab.; 15:606-614. 22560213 10.1016/j.cmet.2012.01.024 PMC3695706

[b33] Newgard C. B. An J. Bain J. R. Muehlbauer M. J. Stevens R. D. Lien L. F. 2009 A branched‐chain amino acid‐related metabolic signature that differentiates obese and lean humans and contributes to insulin resistance. Cell Metab.; 9:311-326. 19356713 10.1016/j.cmet.2009.02.002 PMC3640280

[b34] Pereira S. Marliss E. B. Morais J. A. Chevalier S. Gougeon R. 2008 Insulin resistance of protein metabolism in type 2 diabetes. Diabetes; 57:56-63. 17940118 10.2337/db07-0887

[b35] Pimenta A. S. Gaidhu M. P. Habib S. So M. Fediuc S. Mirpourian M. 2008 Prolonged exposure to palmitate impairs fatty acid oxidation despite activation of AMP‐activated protein kinase in skeletal muscle cells. J. Cell. Physiol.; 217:478-485. 18561258 10.1002/jcp.21520

[b36] Qin L. Q. Xun P. Bujnowski D. Daviglus M. L. Van Horn L. Stamler J. 2011 Higher branched‐chain amino acid intake is associated with a lower prevalence of being overweight or obese in middle‐aged East Asian and Western adults. J. Nutr.; 141:249-254. 21169225 10.3945/jn.110.128520 PMC3021443

[b37] Rui L. Yuan M. Frantz D. Shoelson S. White M. F. 2002 SOCS‐1 and SOCS‐3 block insulin signaling by ubiquitin‐mediated degradation of IRS1 and IRS2. J. Biol. Chem.; 277:42394-42398. 12228220 10.1074/jbc.C200444200

[b38] Schulze M. B. Manson J. E. Willett W. C. Hu F. B. 2003 Processed meat intake and incidence of type 2 diabetes in younger and middle‐aged women. Diabetologia; 46:1465-1473. 14576980 10.1007/s00125-003-1220-7

[b39] Serino A. S. Adegoke O. A. Zargar S. Gordon C. S. Szigiato A. A. Hawke T. J. 2011 Voluntary physical activity and leucine correct impairments in muscle protein synthesis in partially pancreatectomised rats. Diabetologia; 54:3111-3120. 21909838 10.1007/s00125-011-2296-0

[b40] Shepherd P. R. 2005 Mechanisms regulating phosphoinositide 3‐kinase signalling in insulin‐sensitive tissues. Acta Physiol. Scand.; 183:3-12. 15654916 10.1111/j.1365-201X.2004.01382.x

[b41] Solerte S. B. Fioravanti M. Locatelli E. Bonacasa R. Zamboni M. Basso C. 2008 Improvement of blood glucose control and insulin sensitivity during a long‐term (60 weeks) randomized study with amino acid dietary supplements in elderly subjects with type 2 diabetes mellitus. Am. J. Cardiol.; 101:82E-88E. 10.1016/j.amjcard.2008.03.006 18514633

[b42] Song Y. Manson J. E. Buring J. E. Liu S. 2004 A prospective study of red meat consumption and type 2 diabetes in middle‐aged and elderly women: the women's health study. Diabetes Care; 27:2108-2115. 15333470 10.2337/diacare.27.9.2108

[b43] Song M. S. Salmena L. Pandolfi P. P. 2012 The functions and regulation of the PTEN tumour suppressor. Nat. Rev. Mol. Cell Biol.; 13:283-296. 22473468 10.1038/nrm3330

[b44] Thoreen C. C. Chantranupong L. Keys H. R. Wang T. Gray N. S. Sabatini D. M. 2012 A unifying model for mTORC1‐mediated regulation of mRNA translation. Nature; 485:109-113. 22552098 10.1038/nature11083 PMC3347774

[b45] Tremblay F. Lavigne C. Jacques H. Marette A. 2007 Role of dietary proteins and amino acids in the pathogenesis of insulin resistance. Annu. Rev. Nutr.; 27:293-310. 17666010 10.1146/annurev.nutr.25.050304.092545

[b46] Tsugane S. Inoue M. 2010 Insulin resistance and cancer: epidemiological evidence. Cancer Sci.; 101:1073-1079. 20345478 10.1111/j.1349-7006.2010.01521.x PMC11159937

[b47] Um S. H. Frigerio F. Watanabe M. Picard F. Joaquin M. Sticker M. 2004 Absence of S6K1 protects against age‐ and diet‐induced obesity while enhancing insulin sensitivity. Nature; 431:200-205. 15306821 10.1038/nature02866

[b48] Wang X. Hu Z. Hu J. Du J. Mitch W. E. 2006 Insulin resistance accelerates muscle protein degradation: activation of the ubiquitin‐proteasome pathway by defects in muscle cell signaling. Endocrinology; 147:4160-4168. 16777975 10.1210/en.2006-0251

[b49] Wang T. J. Larson M. G. Vasan R. S. Cheng S. Rhee E. P. McCabe E. 2011 Metabolite profiles and the risk of developing diabetes. Nat. Med.; 17:448-453. 21423183 10.1038/nm.2307 PMC3126616

[b50] Westerterp‐Plantenga M. S. Rolland V. Wilson S. A. Westerterp K. R. 1999 Satiety related to 24 h diet‐induced thermogenesis during high protein/carbohydrate vs high fat diets measured in a respiration chamber. Eur. J. Clin. Nutr.; 53:495-502. 10403587 10.1038/sj.ejcn.1600782

[b51] Zargar S. Moreira T. S. Samimi‐Seisan H. Jeganathan S. Kakade D. Islam N. 2011 Skeletal muscle protein synthesis and the abundance of the mRNA translation initiation repressor PDCD4 are inversely regulated by fasting and refeeding in rats. Am. J. Physiol. Endocrinol. Metab.; 300:E986-E992. 21406616 10.1152/ajpendo.00642.2010

